# Longitudinal serum neurofilament light kinetics in post‐anoxic encephalopathy

**DOI:** 10.1002/acn3.51903

**Published:** 2023-09-25

**Authors:** Giulio Disanto, Michele Villa, Aleksandra Maleska Maceski, Chiara Prosperetti, Claudio Gobbi, Jens Kuhle, Tiziano Cassina, Pamela Agazzi

**Affiliations:** ^1^ Neurocenter of Southern Switzerland, Civic Hospital, Ente Ospedaliero Cantonale Lugano Switzerland; ^2^ Department of Cardiac Anesthesia and Intensive Care Cardiocentro Ticino Institute, Ente Ospedaliero Cantonale Lugano Switzerland; ^3^ Department of Neurology University Hospital and University of Basel Basel Switzerland; ^4^ Multiple Sclerosis Centre and Research Centre for Clinical Neurimmunology and Neuroscience (RC2NB), Departments of Biomedicine and Clinical Research University Hospital and University of Basel Basel Switzerland

## Abstract

Serum neurofilament light (sNfL) is a promising marker of outcome after cardiac arrest, but its kinetics are unclear. We prospectively measured sNfL concentrations in 62 patients at 0, 1, 3, 5, 7 and 10 days after cardiac arrest. Survivors and non‐survivors had similar sNfL at admission (14.2 [8.6–21.9] vs. 22.5 [14.2–46.9] pg/mL) but largely different at 24 h (16.4 [10.2–293] vs. 464.3 [151.8–1658.2], respectively). The AUC for sNfL concentrations predicting death was above 0.95 from Day 1 to 10 (highest on Day 3). Late sNfL measurements may exert prognostic value, especially when early samples are unavailable or prognosis remains unclear.

## Introduction

Prognosis of post‐anoxic encephalopathy after cardiac arrest (CA) currently integrates clinical findings, somatosensory evoked potentials, electroencephalogram (EEG), neuroimaging and serum neuron‐specific enolase (NSE) concentration.^1^ The value of NSE is unfortunately limited to the first 72 h after CA^1^, ^2^ and susceptible to confounding factors such as haemolysis.^3^, ^4^ Studies have shown that serum neurofilament light (sNfL) concentrations measured up to 72 h after CA are more accurate predictors of neurologic outcome than NSE.^5^ Some preliminary data from our group suggested that sNfL concentrations may remain high at later time points,^6^ but sNfL kinetics are still unclear. This is relevant as mortality is often delayed, and no late serum prognostic markers are available. We therefore aimed to investigate longitudinal sNfL kinetics in patients suffering from post‐anoxic encephalopathy up to 10 days after CA, and to suggest potential time‐specific sNfL cut‐offs associated with both death and favourable clinical outcomes.

## Methods

We prospectively recruited all consecutive patients with post‐anoxic encephalopathy due to CA admitted to Cardiocentro Ticino Institute (Lugano, Switzerland) between September 2020 and August 2022. Inclusion criteria were (1) age above 18 years; (2) evidence of CA by ventricular tachycardia, ventricular fibrillation, pulseless electrical activity or asystole; (3) informed consents from patients or legal representatives. Exclusion criteria were (1) known underlying neurodegenerative disease, (2) pregnancy, (3) CA due to trauma and (4) concomitant acute stroke.

Serum samples were collected at time of admission and at 1, 3, 5, 7 and 10 days after CA. sNfL concentrations were measured in duplicate with the NF‐light assay (Quanterix, Billerica, MA, USA) as previously described.^7^ Standard 10–20 montage EEGs were also registered when clinically required. Outcomes were mortality and cerebral performance category (CPC; 1 = *normal/good cerebral performance*; 2 = *disabled but independent*; 3 = *conscious but disabled and dependent*; 4 = *unconscious, coma or vegetative state*; 5 = *death*) at hospital discharge and at 6 months after CA. Information on outcomes at 6 months were retrieved by phone interviews.

Variables were described by median and interquartile range (IQR) or counts and percentage, as appropriate. Multivariable mixed‐effect models with individuals as random effect were used to simultaneously test variables for association with log‐transformed sNfL concentrations measured at all time points. Regression coefficients were back‐transformed to the original scale to allow easier interpretation of coefficients. Predicting power and sNfL cut‐offs for mortality and survival were investigated using receiver operating characteristic (ROC). ROC analyses were performed first using all available samples collected in the study, and then using only those samples collected from patients who had not regained full consciousness (defined as reduced FOUR scale^8^) and were still in intensive care unit (ICU) at time of sampling. All analyses were performed using the statistical software R. The study was approved by Canton Ticino ethics committee (CE‐TI3639). This report follows the STROBE guidelines. According to Swiss regulations, the study was centrally registered in BASEC (Business Administration System for Ethics Committees).

## Results

A total of 62 CA patients (age = 66.5 [56.0–80.8], males = 45 [72.6%]) were included in the study. Acute coronary syndromes (ACS) were the most frequent cause of CA (*n* = 48 [77.4%]). Thirty‐three patients (53.2%) were alive at 6 months (24 with CPC 1 and 9 with CPC 2). Twenty‐eight (45.2%) patients died before hospital discharge, and one 4 months after discharge because of an additional cardiovascular event. Life‐sustaining therapies were withdrawn in 18 individuals (14 because of poor neurological outcome and 4 because of multi‐organ failure). Fifty‐one (82.2%) had ≥2, 45 (72.6%) had ≥3, 41 (66.1%) had ≥4, 34 (54.8%) had ≥5 and 19 (30.6%) had 6 samples collected (total samples *n* = 252). Main demographic and clinical characteristics are shown in Table 1.

**Table 1:** Demographic and clinical variables at baseline, during hospitalization and clinical outcome in all cardiac arrest patients included in the study, non‐survivors and survivors at discharge.

Variables	All (*n* = 62)	Dead at hospital discharge (*n* = 28)	Alive at hospital discharge (*n* = 34)
Age			
Years	66.5 (56.0–80.8)	71.5 (60.3–82.3)	62.0 (55.0–72.8)
Gender			
Male	45 (72.6)	21 (75.0)	24 (70.6)
Witnessed arrest			
Yes	55 (88.7)	24 (85.7)	31 (91.2)
First rhythm			
Shockable	53 (85.5)	20 (71.4)	33 (97.1)
Time to ROSC (min)	26.5 (17.3–41.5)	32.5 (25.0–47.5)	22.0 (11.5–33.8)
Arrest cause			
ACS	48 (77.4)	21 (75.0)	27 (79.4)
Arrhythmia	10 (16.1)	3 (10.7)	7 (20.6)
Hypoxia	3 (4.8)	3 (10.7)	0 (0.0)
Pulmonary embolism	1 (1.6)	1 (3.6)	0 (0.0)
Mechanical ventilation			
Yes	58 (93.5)	28 (100)	30 (88.2)
Day 1 lactate (mmol/L)	1.6 (1.2–2.8)	1.6 (1.4–3.6)	1.6 (1.0–2.3)
Day 1 non‐neurological SOFA	6 (5–9)	8 (6–10)	5 (3–8)
EEG			
Slow background	13 (21.0)	3 (10.7)	10 (29.4)
GPD	6 (9.7)	6 (21.4)	0 (0)
Suppression	5 (8.1)	5 (17.9)	0 (0)
Burst‐suppression	3 (4.8)	3 (10.7)	0 (0)
Not performed	35 (56.5)	11 (39.3)	24 (70.6)
NSE at 24 h (38 patients) (μg/L	43.4 (22.2–64.6)	62.5 (44.8–98.22)	22.3 (19.4–44.6)
NSE at 48–72 h (36 patients) (μg/L)	41.4 (16.0–92.7)	89.6 (60.5–236.0)	17.2 (14.2–36.7)
Sensory evoked potentials			
Present	7 (11.3%)	6 (21.4)	1 (2.9)
Absent	7 (11.3%)	7 (25.0)	0 (0.0)
Not performed	48 (77.4)	15 (53.6)	33 (97.1)
Diffuse anoxic damage on brain CT/MRI			
Present	7 (11.3)	6 (21.4)	1 (2.9)
Absent	15 (24.2)	2 (7.1)	13 (38.2)
Not performed	40 (64.5)	20 (71.4)	20 (58.8)
CPC at discharge			
1	17 (27.4)	0 (0)	17 (50.0)
2	11 (17.7)	0 (0)	11 (32.4)
3	6 (9.7)	0 (0)	6 (17.6)
4	0	0 (0)	0 (0)
5	28 (45.2)	28 (100)	0 (0)
CPC at 6 months			
1	24 (38.7)	0 (0)	24 (70.6)
2	9 (14.5)	0 (0)	9 (26.5)
3	0 (0)	0 (0)	0 (0)
4	0 (0)	0 (0)	0 (0)
5	29 (46.8)	28 (100)	1 (2.9)
Number of samples collected			
1	11 (17.7)	10 (35.7)	1 (2.9)
2	6 (9.7)	5 (17.9)	1 (2.9)
3	4 (6.5)	3 (10.7)	1 (2.9)
4	7 (11.3)	4 (14.3)	3 (8.8)
5	15 (24.2)	2 (7.1)	13 (38.2)
6	19 (30.6)	4 (14.3)	15 (44.1)

ACS, acute coronary syndrome; CPC, cerebral performance category; GPD, generalized periodic discharges; NSE, neuronal‐specific enolase; ROSC, return of spontaneous circulation; SOFA, Sequential Organ Failure Assessment.

**Figure 1:**
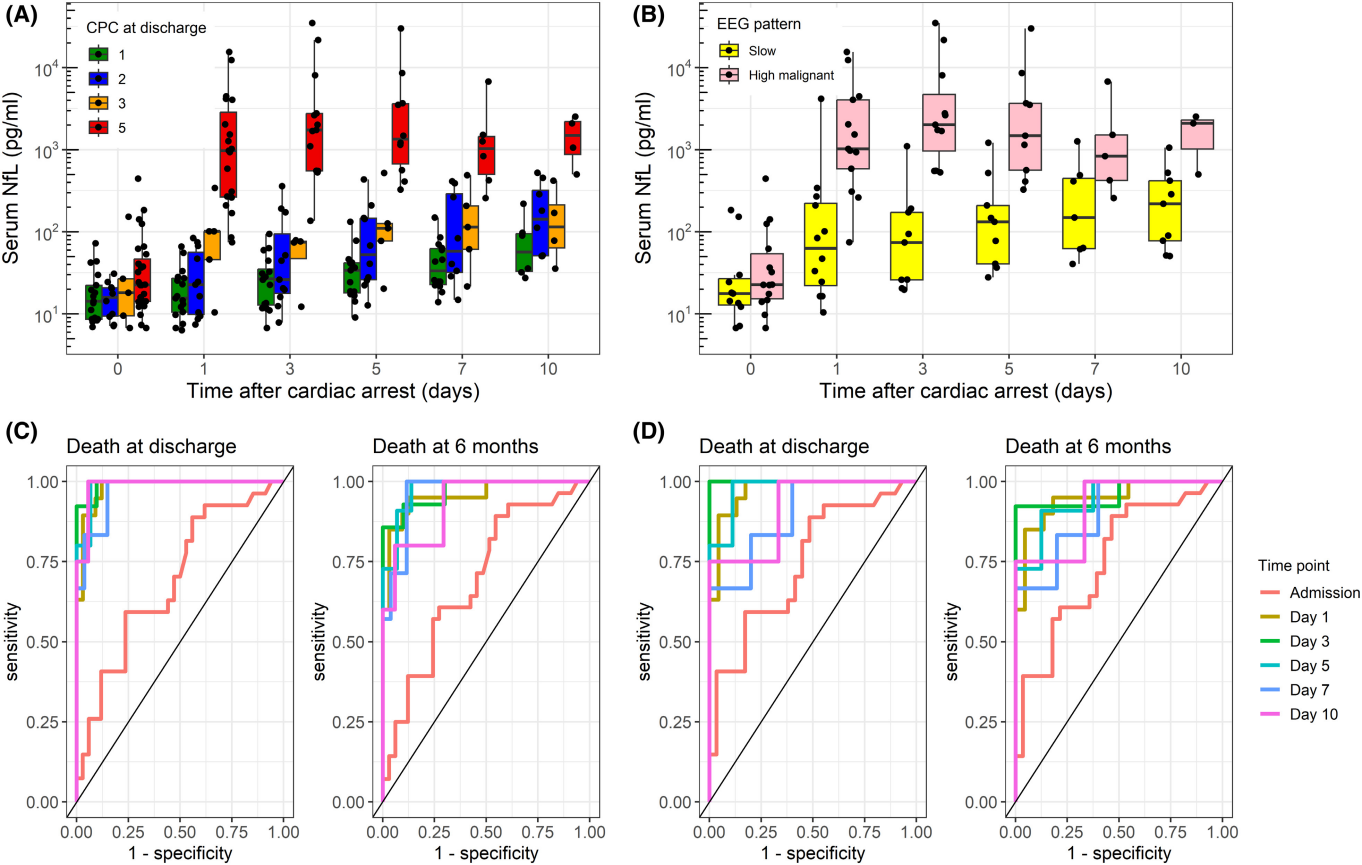
(A) Increasing sNfL concentrations from admission to up to 10 days after cardiac arrest, stratified by cerebral performance category (CPC) at hospital discharge; (B) Longitudinal changes in sNfL concentrations in patients with favourable (slow background) and highly malignant EEG patterns; (C) Time‐specific receiver operating characteristic (ROC) curves for sNfL concentrations predicting mortality at discharge (left) and at 6 months after cardiac arrest (right), using all available samples; (D) Time‐specific ROC curves for sNfL concentrations predicting mortality at discharge (left) and at 6 months after cardiac arrest (right), using only samples collected from patients who were not full conscious and still in the ICU at time of sampling.

Differences in sNfL levels between survivors and non‐survivors at hospital discharge were numerically small at admission (14.2 [8.6–21.9] vs. 22.5 [14.2–46.9] pg/mL, respectively), while significant at 24 h (16.4 [10.2–293] vs. 464.3 [151.8–1,658.2] pg/mL, respectively, Fig. 1A). Similar figures were obtained when considering clinical outcomes at 6 months after CA (Figure S1).

Using mixed effect models, sNfL concentrations were positively associated with age (*β* = 1.02, 95% CI = 1.00–1.04, *P* = 0.036), day of sampling since CA (*β* = 1.25, 95% CI = 1.20–1.31, *P* < 0.001) and CPC at hospital discharge (*β* = 2.13, 95% CI = 1.80–2.53, *P* < 0.001, Table S1). Among patients with EEG (*n* = 27), those with highly malignant patterns (i.e. burst‐suppression or suppression with or without generalized periodic discharges)^9^ had higher sNfL than slow backgrounds (*β* = 8.22, 95% CI = 3.48–18.72, *P* < 0.001, Fig. 1B, Table S2).

When predicting mortality at hospital discharge using all samples, the area under the curve (AUC) for sNfL was above 0.95 from Day 1 to 10, and the highest on Day 3 (AUC = 0.99, 95% CI = 0.98–1.00; sNfL cut‐off with 100% specificity = 444.7 pg/mL, Fig. 1C). When predicting survival at hospital discharge, the sNfL cut‐off on Day 3 below which there was 100% specificity for survival was 114.6 pg/mL (Table 2, upper part). The same analysis was repeated using only samples collected from patients who were not full conscious and still in the ICU (Figure S2), with similar results in terms of outcome prediction (Fig. 1D and Table 2 lower part). The AUC for 6 months mortality and survival were slightly lower but still above 0.90 from Day 1 to 10. Time specific AUC and cut‐offs with 100% specificity for mortality and survival at 6 months after CA are presented in Table S3.

**Table 2:** Time‐specific area under the curve (AUC) with 95% confidence intervals (CI) for sNfL predicting outcome at hospital discharge, with sNfL cut‐offs with 100% specificity for in‐hospital death and survival (and corresponding sensitivities).

	AUC	95% CI	Cut‐off with 100% specificity for mortality	Corresponding sensitivity	Cut‐off with 100% specificity for survival	Corresponding sensitivity
NfL time point (all available samples)						
Admission	0.692	0.557–0.826	sNfL >168.1	7%	sNfL <5.55	6%
Day 1	0.981	0.953–1.000	sNfL >464.3	63%	sNfL <74.2	88%
Day 3	0.993	0.976–1.000	sNfL >444.7	92%	sNfL <114.6	90%
Day 5	0.987	0.962–1.000	sNfL >540.5	80%	sNfL <267.8	93%
Day 7	0.969	0.913–1.000	sNfL >659.7	67%	sNfL <232.1	85%
Day 10	0.986	0.948–1.000	sNfL >790.2	75%	sNfL <476.7	94%
NfL time point (only samples in reduced consciousness)						
Admission	0.745	0.614–0.875	sNfL >98.7	15%	sNfL <5.55	7%
Day 1	0.972	0.933–1.000	sNfL >464.3	63%	sNfL <74.2	83%
Day 3	1.000	1.000–1.000	sNfL >360.5	100%	sNfL <360.5	100%
Day 5	0.978	0.925–1.000	sNfL >540.5	80%	sNfL <267.8	89%
Day 7	0.900	0.713–1.000	sNfL >659.7	67%	sNfL <232.1	60%
Day 10	0.917	0.686–1.000	sNfL >790.2	75%	sNfL <460.1	67%

The upper part of the tables shows results using all available collected samples. The lower part of the table shows results using only samples collected from patients who were not full conscious and still in the ICU.

We looked at cut‐offs predicting survival with good outcomes (CPC 1–2) at 6 months after CA with 100% sensitivity. As all individuals who were alive at 6 months after CA had CPC 1–2, sNfL cut‐offs with 100% sensitivity for CPC 1–2 mirrored those with 100% specificity for mortality at 6 months (Table S4).

## Discussion

We describe longitudinal sNfL variation in post‐anoxic encephalopathy up to 10 days after CA. Higher sNfL levels were associated with age, suggesting that age‐related brain susceptibility to the anoxic insult can contribute to neuronal death. Although EEG were only performed in a minority of patients, we confirmed that malignant EEG patterns reflect the extent of brain injury as measured by sNfL.^10^

While sNfL levels were comparable in survivors and non‐survivors at admission, non‐survivors showed up to 1000‐fold sNfL increase at 24 h, reaching higher concentrations than those observed in other conditions affecting the central nervous system (including most neurodegenerative diseases).^11^ Notably, sNfL concentrations remained high for up to 10 days after CA. This agrees with other studies showing that sNfL levels return to baseline only several months after central nervous system damage.^12^ This represents a great advantage as compared to NSE, and makes sNfL the first potential late serum marker of outcome in post‐anoxic encephalopathy.

Accordingly, the predictive power of sNfL was greater within 24–72 h, but later measurements also showed AUC above 0.90. We provide approximate time‐specific sNfL cut‐offs with 100% specificity for mortality and survival. As an example, in this cohort, sNfL concentrations approximately above 400 pg/mL on Day 3 after CA were specific predictors of death (both in the overall and in the sensitivity analysis using only individuals who had not regained full consciousness). Our cut‐offs were relatively lower compared to those provided by Moseby‐Knappe et al.,^13^ which represents the largest study available to date. This discrepancy can be likely interpreted as a consequence of the different sample size.

As sNfL concentrations are positively associated with age, it has been suggested to use age‐corrected thresholds (e.g. percentiles or Z scores) to define normal and pathological sNfL values.^7^ All our analyses were corrected by age, but when defining sNfL cut‐offs, we preferred to use the raw unadjusted concentrations. Nonetheless, as sNfL rarely reach concentrations above 50 pg/mL (even in older individuals), the extremely high sNfL concentrations seen in post‐anoxic patients make any correction by age less relevant. We also preferred to use original sNfL concentrations to make our results easier to be interpreted and compared to other studies.

Strengths of the study are represented by the standardized and prospective collection of samples at specific time points from consecutively admitted CA patients, including very early (admission) and late (10 days) samples, that are not available in most of the studies performed to date. The main limitation is the relatively small sample size of the cohort, which suggests caution in the application of prognostic sNfL cut‐offs. In particular, when the analysis was restricted to patients who had not regained full consciousness and were still in the ICU, the sample size became too small to make our cut‐offs clinically applicable. The relatively high proportion of patients with shockable rhythms may also limit generalizability of findings. Prognostication assessments (EEG, sensory evoked potentials, NSE concentrations and brain imaging) were performed when deemed indicated based on the clinical situation (therefore in most but not all patients), without strictly following Nolan et al guidelines.^14^

To conclude, we provide new information regarding longitudinal kinetics of sNfL in post‐anoxic encephalopathy patients up to 10 days after the anoxic insult, that can be used as a guide when planning future studies. Based on these data, we suggest that sNfL measurements performed >72 h after CA could also be of value, especially when early samples/NSE are unavailable or prognosis remains unclear. We provide approximate time‐specific cut‐offs with 100% specificity for death and survival, to be integrated and replicated in future studies of larger sample size.

## Conflicts of Interest

Giulio Disanto reports no conflicts of interest. Michele Villa reports no conflicts of interest. Aleksandra Maleska Maceski reports no conflicts of interest. Chiara Prosperetti reports no conflicts of interest. Claudio Gobbi reports that the employer (Department of Neurology, Regional Hospital Lugano (EOC), Lugano, Switzerland) receives financial support from Teva, Merck Serono, Biogen Idec, Bayer Schering, Genzyme, Roche and Novartis. The submitted work is not related to these agreements. Jens Kuhle served on scientific advisory boards for Novartis Pharmaceuticals, Merck, Biogen, Sanofi Genzyme, Roche and Bayer; has received funding for travel and/or speaker honoraria from Biogen, Sanofi Genzyme, Novartis, Merck Serono, Roche, Teva and the Swiss MS Society; and research support from Bayer, Biogen, Merck, Sanofi Genzyma, Novartis, Roche, ECTRIMS Research Fellowship Programme, University of Basel, Swiss MS Society, Swiss National Research Foundation (320030_160221). Tiziano Cassina reports no conflicts of interest. Pamela Agazzi reports no conflicts of interest.

## Author Contributions

Giulio Disanto: conception and design of the study; acquisition and analysis of data; drafting a significant portion of the manuscript. Michele Villa: acquisition and analysis of data. Aleksandra Maleska Maceski: acquisition and analysis of data. Chiara Prosperetti; conception and design of the study; drafting a significant portion of the manuscript. Claudio Gobbi: conception and design of the study. Jens Kuhle: acquisition and analysis of data; drafting a significant portion of the manuscript. Tiziano Cassina: conception and design of the study; acquisition and analysis of data; drafting a significant portion of the manuscript. Pamela Agazzi: conception and design of the study; acquisition and analysis of data; drafting a significant portion of the manuscript.

## Supporting information


**Figure S1.** Increasing sNfL concentrations from admission to up to 10 days after cardiac arrest, stratified by cerebral performance category (CPC) at 6 months after cardiac arrest.
**Table S1.** Multivariable mixed‐effects regression testing the association between variables of interest with log‐transformed sNfL (predicted variable). Regression coefficients are back‐transformed to the original scale (e.g. *β* for age = 1.02 indicates that for every one‐unit increase in age, sNfL increases by about 2%). ROSC, return of spontaneous circulation; SOFA, Sequential Organ Failure Assessment.
**Table S2.** Multivariable mixed‐effect regression testing the association between EEG pattern (highly malignant vs slow background) and longitudinal sNfL (predicted variable). Regression coefficients are back‐transformed to the original scale.
**Figure S2.** Increasing sNfL concentrations from admission to up to 10 days after cardiac arrest stratified by survival at hospital discharge, using only samples collected from patients who were not fully conscious and still in the ICU at time of sampling. By doing this, we excluded from the analysis 5 individuals since admission, 6 since day 1, 12 since day 3, 2 since day 5, 3 since day 7 and 3 since day 10.
**Table S3.** Time specific area under the curve (AUC) with 95% confidence intervals (CI) for sNfL predicting outcome at 6 months after cardiac arrest, with sNfL cutoffs with 100% specificity for death and survival (and corresponding sensitivities). The upper part of the table shows results using all available collected samples. The lower part of the table shows results using only samples collected from patients who were not fully conscious and still in the ICU.
**Table S4.** Time specific area under the curve (AUC) with 95% confidence intervals (CI) for sNfL predicting CPC 1–2 at 6 months after cardiac arrest, with sNfL cutoffs with 100% sensitivity and corresponding specificities.

## References

[1] Rossetti AO , Rabinstein AA , Oddo M . Neurological prognostication of outcome in patients in coma after cardiac arrest. Lancet Neurol. 2016;15(6):597‐609. 27017468 10.1016/S1474-4422(16)00015-6

[2] Fogel W , Krieger D , Veith M , et al. Serum neuron‐specific enolase as early predictor of outcome after cardiac arrest. Crit Care Med. 1997;25(7):1133‐1138. 9233737 10.1097/00003246-199707000-00012

[3] Sandroni C , D'Arrigo S , Nolan JP . Prognostication after cardiac arrest. Crit Care. 2018;22(1):150. 29871657 10.1186/s13054-018-2060-7 PMC5989415

[4] Ramont L , Thoannes H , Volondat A , Chastang F , Millet MC , Maquart FX . Effects of hemolysis and storage condition on neuron‐specific enolase (NSE) in cerebrospinal fluid and serum: implications in clinical practice. Clin Chem Lab Med. 2005;43(11):1215‐1217. 16232088 10.1515/CCLM.2005.210

[5] Hoiland RL , Rikhraj KJK , Thiara S , et al. Neurologic prognostication after cardiac arrest using brain biomarkers: a systematic review and meta‐analysis. JAMA Neurol. 2022;79(4):390‐398. 35226054 10.1001/jamaneurol.2021.5598 PMC8886448

[6] Disanto G , Prosperetti C , Gobbi C , et al. Serum neurofilament light chain as a prognostic marker in postanoxic encephalopathy. Epilepsy Behav. 2019;101:106432. 31375414 10.1016/j.yebeh.2019.07.033

[7] Benkert P , Meier S , Schaedelin S , et al. Serum neurofilament light chain for individual prognostication of disease activity in people with multiple sclerosis: a retrospective modelling and validation study. Lancet Neurol. 2022;21(3):246‐257. 35182510 10.1016/S1474-4422(22)00009-6

[8] Wijdicks EFM , Bamlet WR , Maramattom BV , Manno EM , McClelland RL . Validation of a new coma scale: the FOUR score. Ann Neurol. 2005;58(4):585‐593. 16178024 10.1002/ana.20611

[9] Westhall E , Rossetti AO , van Rootselaar A‐F , et al. Standardized EEG interpretation accurately predicts prognosis after cardiac arrest. Neurology. 2016;86(16):1482‐1490. 26865516 10.1212/WNL.0000000000002462 PMC4836886

[10] Grindegård L , Cronberg T , Backman S , et al. Association between EEG patterns and serum Neurofilament light after cardiac arrest. Neurology. 2022;98(24):e2487‐e2498. 35470143 10.1212/WNL.0000000000200335 PMC9231840

[11] Yuan A , Nixon RA . Neurofilament proteins as biomarkers to monitor neurological diseases and the efficacy of therapies. Front Neurosci. 2021;15:689938. 34646114 10.3389/fnins.2021.689938 PMC8503617

[12] Bergman J , Dring A , Zetterberg H , et al. Neurofilament light in CSF and serum is a sensitive marker for axonal white matter injury in MS. Neurol Neuroimmunol Neuroinflammation. 2016;3(5):e271. 10.1212/NXI.0000000000000271 PMC4972001 27536708

[13] Moseby‐Knappe M , Mattsson N , Nielsen N , et al. Serum neurofilament light chain for prognosis of outcome after cardiac arrest. JAMA Neurol. 2018;76:64. 10.1001/jamaneurol.2018.3223 PMC6440255 30383090

[14] Nolan JP , Sandroni C , Böttiger BW , et al. European resuscitation council and European society of intensive care medicine guidelines 2021: post‐resuscitation care. Resuscitation. 2021;161:220‐269. 33773827 10.1016/j.resuscitation.2021.02.012

