# Supplementary material for: Longitudinal serum neurofilament light kinetics in post‐anoxic encephalopathy
Source: Ann Clin Transl Neurol. 2023 Sep 25;10(12):2407–12. doi: 10.1002/acn3.51903 (PMC10723239; doi:10.1002/acn3.51903)
Supplement: Supplementary file 1 — Figure S1. Increasing sNfL concentrations from admission to up to 10 days after cardiac arrest, stratified by cerebral performance category (CPC) at 6 months after cardiac arrest. Table S1. Multivariable mixed‐effects regression testing the association between variables of interest with log‐transformed sNfL (predicted variable). Regression coefficients are back‐transformed to the original scale (e.g. β for age = 1.02 indicates that for every one‐unit increase in age, sNfL increases by about 2%). ROSC, return of spontaneous circulation; SOFA, Sequential Organ Failure Assessment. Table S2. Multivariable mixed‐effect regression testing the association between EEG pattern (highly malignant vs slow background) and longitudinal sNfL (predicted variable). Regression coefficients are back‐transformed to the original scale. Figure S2. Increasing sNfL concentrations from admission to up to 10 days after cardiac arrest stratified by survival at hospital discharge, using only samples collected from patients who were not fully conscious and still in the ICU at time of sampling. By doing this, we excluded from the analysis 5 individuals since admission, 6 since day 1, 12 since day 3, 2 since day 5, 3 since day 7 and 3 since day 10. Table S3. Time specific area under the curve (AUC) with 95% confidence intervals (CI) for sNfL predicting outcome at 6 months after cardiac arrest, with sNfL cutoffs with 100% specificity for death and survival (and corresponding sensitivities). The upper part of the table shows results using all available collected samples. The lower part of the table shows results using only samples collected from patients who were not fully conscious and still in the ICU. Table S4. Time specific area under the curve (AUC) with 95% confidence intervals (CI) for sNfL predicting CPC 1–2 at 6 months after cardiac arrest, with sNfL cutoffs with 100% sensitivity and corresponding specificities. [file ACN3-10-2407-s001.docx]

**SUPPLEMENTARY MATERIAL**

**Supplementary figure 1:** Increasing sNfL concentrations from admission to up to 10 days after cardiac arrest, stratified by cerebral performance category (CPC) at 6 months after cardiac arrest.


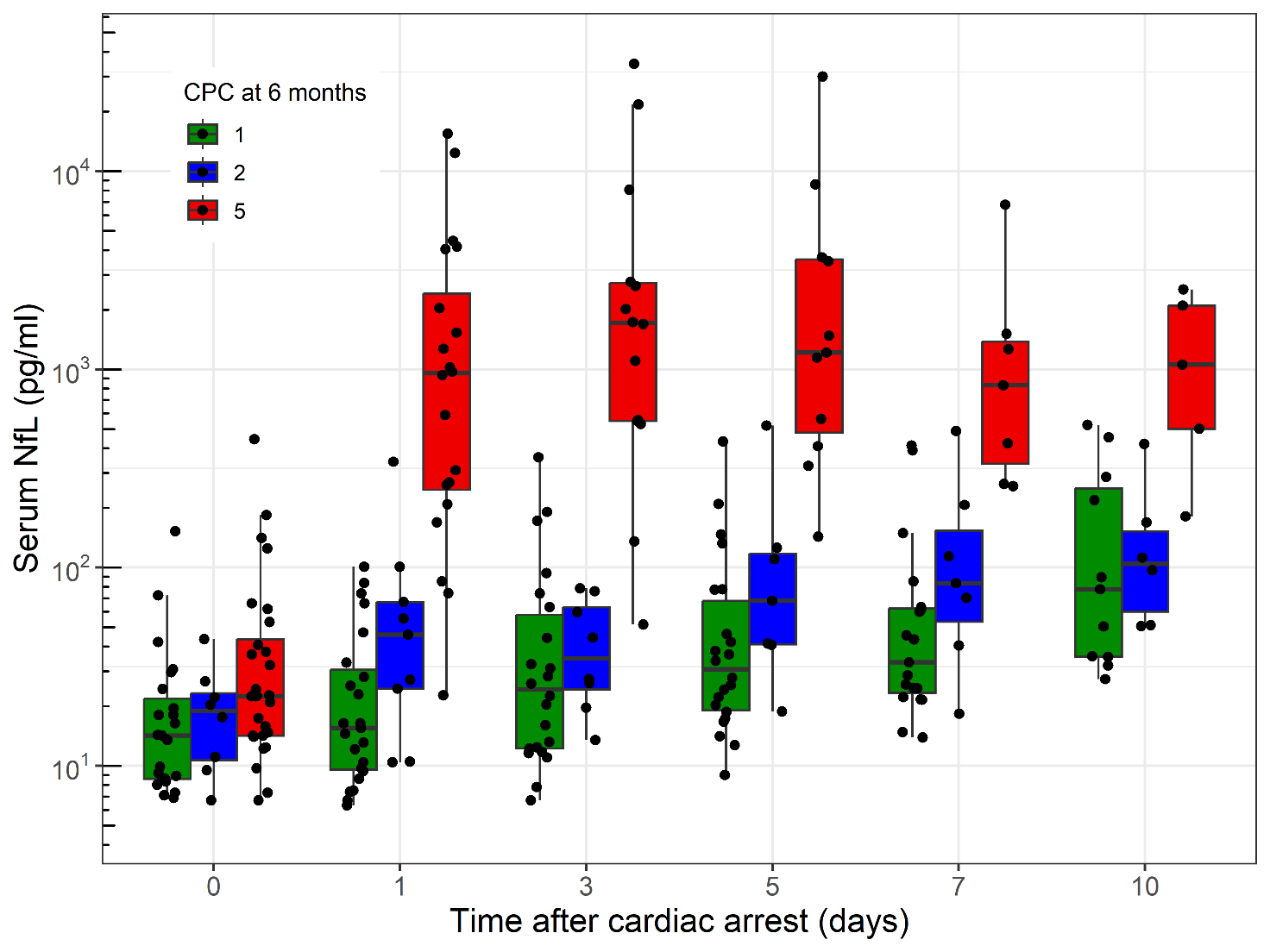


**Supplementary table 1:** Multivariable mixed-effects regression testing the association between variables of interest with log-transformed sNfL (predicted variable). Regression coefficients are back-transformed to the original scale (e.g. β for age=1.02 indicates that for every one-unit increase in age, sNfL increases by about 2%). SOFA=Sequential Organ Failure Assessment; ROSC=return of spontaneous circulation.

| **Fixed effects** | **Beta** | **95%CI** | **P-value** |
| --- | --- | --- | --- |
| Age (per year) | 1.02 | 1.00 - 1.04 | 0.036 |
| Gender (M vs F) | 0.88 | 0.47 - 1.65 | 0.688 |
| Days since cardiac arrest | 1.25 | 1.20 - 1.31 | <0.001 |
| CPC at hospital discharge | 2.13 | 1.80 – 2.53 | <0.001 |
| Day 1 non-neurological SOFA | 1.04 | 0.94 - 1.15 | 0.443 |
| Day 1 lactate | 1.00 | 0.91 - 1.10 | 0.968 |
| Time to ROSC | 1.00 | 0.99 - 1.02 | 0.587 |

**Supplementary table 2:** Multivariable mixed-effect regression testing the association between EEG pattern (highly malignant vs slow background) and longitudinal sNfL (predicted variable). Regression coefficients are back-transformed to the original scale.

| **Fixed effects** | **Beta** | **95%CI** | **P-value** |
| --- | --- | --- | --- |
| Age | 0.98 | 0.95 - 1.01 | 0.234 |
| Gender (M vs F) | 1.68 | 0.62 - 4.56 | 0.298 |
| EEG (highly malignant vs  slow background) | 8.22 | 3.48 - 18.72 | <0.001 |

**Supplementary figure 2:** Increasing sNfL concentrations from admission to up to 10 days after cardiac arrest stratified by survival at hospital discharge, using only samples collected from patients who were not fully conscious and still in the ICU at time of sampling. By doing this, we excluded from the analysis 5 individuals since admission, 6 since day 1, 12 since day 3, 2 since day 5, 3 since day 7 and 3 since day 10.

**
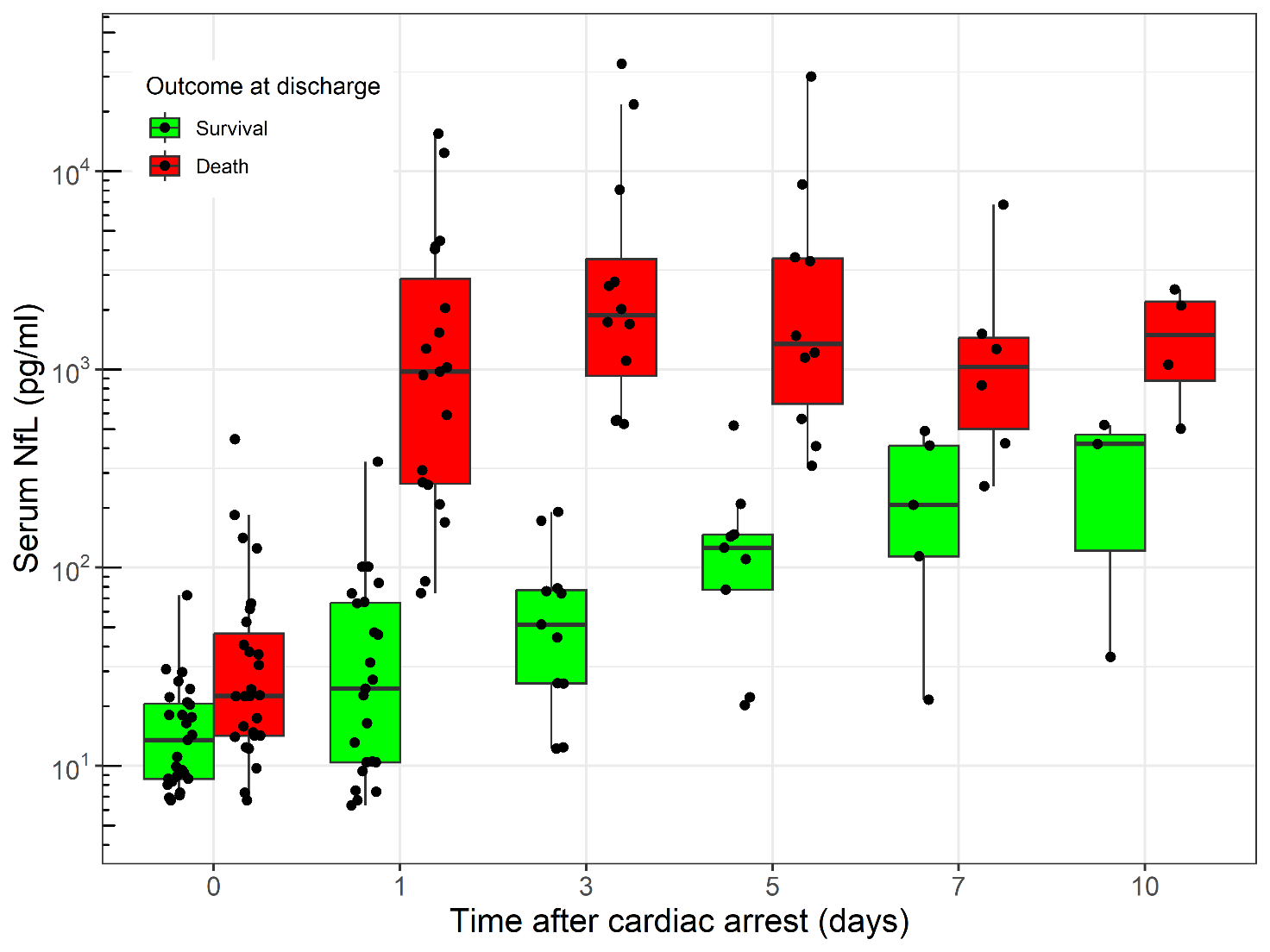
**

**Supplementary table 3:** Time specific area under the curve (AUC) with 95% confidence intervals (CI) for sNfL predicting outcome at 6 months after cardiac arrest, with sNfL cutoffs with 100% specificity for death and survival (and corresponding sensitivities). The upper part of the tables shows results using all available collected samples. The lower part of the table shows results using only samples collected from patients who were not fully conscious and still in the ICU.

| **NfL time point (all available samples)** | **AUC** | **95%CI** | **Cutoff with 100% specificity for mortality** | **Corresponding sensitivity** | **Cutoff with 100% specificity for survival** | **Corresponding sensitivity** |
| --- | --- | --- | --- | --- | --- | --- |
| **Admission** | 0.696 | 0.563-0.829 | sNfL > 168.1 | 7% | sNfL < 5.5 | 6% |
| **Day 1** | 0.956 | 0.900-1.000 | sNfL > 464.3 | 60% | sNfL < 19.5 | 50% |
| **Day 3** | 0.971 | 0.925-1.000 | sNfL > 444.7 | 86% | sNfL < 47.9 | 70% |
| **Day 5** | 0.975 | 0.937-1.000 | sNfL > 540.5 | 73% | sNfL < 137.6 | 86% |
| **Day 7** | 0.961 | 0.903-1.000 | sNfL > 659.7 | 57% | sNfL < 232.1 | 88% |
| **Day 10** | 0.929 | 0.804-1.000 | sNfL > 790.2 | 60% | sNfL < 175.1 | 71% |
| **NfL time point (only samples in reduced consciousness)** | **AUC** | **95%CI** | **Cutoff with 100% specificity for mortality** | **Corresponding sensitivity** | **Cutoff with 100% specificity for survival** | **Corresponding sensitivity** |
| **Admission** | 0.751 | 0.623-0.880 | sNfL > 98.7 | 14% | sNfL < 5.5 | 7% |
| **Day 1** | 0.945 | 0.878-1.000 | sNfL > 464.3 | 60% | sNfL < 19.5 | 45% |
| **Day 3** | 0.961 | 0.882-1.000 | sNfL > 360.5 | 92% | sNfL < 47.9 | 50% |
| **Day 5** | 0.943 | 0.847-1.000 | sNfL > 540.5 | 73% | sNfL < 134.5 | 63% |
| **Day 7** | 0.900 | 0.713-1.000 | sNfL > 659.7 | 67% | sNfL < 232.1 | 60% |
| **Day 10** | 0.917 | 0.686-1.000 | sNfL > 790.2 | 75% | sNfL < 460.1 | 67% |

**Supplementary table 4:** Time specific area under the curve (AUC) with 95% confidence intervals (CI) for sNfL predicting CPC 1-2 at 6 months after cardiac arrest, with sNfL cutoffs with 100% sensitivity and corresponding specificities.

| **NfL time point (all available samples)** | **AUC** | **95%CI** | **Cutoff with 100% sensitivity for CPC 1-2** | **Corresponding specificity** |
| --- | --- | --- | --- | --- |
| **Admission** | 0.696 | 0.563-0.829 | sNfL < 168.1 | 7% |
| **Day 1** | 0.956 | 0.900-1.000 | sNfL < 464.3 | 60% |
| **Day 3** | 0.971 | 0.925-1.000 | sNfL < 444.7 | 86% |
| **Day 5** | 0.975 | 0.937-1.000 | sNfL < 540.5 | 73% |
| **Day 7** | 0.961 | 0.903-1.000 | sNfL < 659.7 | 57% |
| **Day 10** | 0.929 | 0.804-1.000 | sNfL < 790.2 | 60% |
